# Supplementary material for: Effects of Diabetes Quality Assessment on Diabetes Management Behaviors Based on a Nationwide Survey
Source: Int J Environ Res Public Health. 2022 Nov 27;19(23):15781. doi: 10.3390/ijerph192315781 (PMC9740040; doi:10.3390/ijerph192315781)
Supplement: Supplementary file 1 [file ijerph-19-15781-s001.zip › ijerph-1958997-supplementary.pdf]

Supplementary Materials

Table S1. Multilevel multivariate logistic regression for current smoking status in male diabetes patients

	Model 0	Model 1	Model 2	Model 3	Model 4
**Fixed effect**					
**Individuals level**					
**Age (10 years)**		0.96 (0.95–0.96)	0.95 (0.95–0.96)	0.95 (0.95–0.95)	0.95 (0.95–0.95)
**Drinking behavior**					
**Lifetime abstainers**			1 (reference)	1 (reference)	1 (reference)
**Ex-drinkers**			0.87 (0.72–1.05)	0.88 (0.73–1.06)	0.88 (0.73–1.07)
**Occasional drinkers**			1.27 (1.04–1.56)	1.31 (1.06–1.61)	1.32 (1.07–1.62)
**Current drinkers**			1.90 (1.61–2.25)	1.97 (1.66–2.34)	1.98 (1.67–2.35)
**Walking activity**[1]			0.84 (0.77–0.91)	0.86 (0.79–0.93)	0.86 (0.79–0.93)
**Poor self-rated health**			1.21 (1.15–1.27)	1.17 (1.11–1.24)	1.17 (1.11–1.24)
**Body mass index (kg/m^2)**					
**< 18.5**			1.30 (0.96–1.77)	1.26 (0.93–1.73)	1.28 (0.93–1.74)
**18.5–22.9**			1 (reference)	1 (reference)	1 (reference)
**23.0–24.9**			0.66 (0.59–0.74)	0.66 (0.59–0.74)	0.66 (0.59–0.74)
**25.0–29.9**			0.55 (0.49–0.61)	0.55 (0.50–0.62)	0.56 (0.50–0.62)
**≥ 30.0**			0.41 (0.34–0.48)	0.40 (0.34–0.48)	0.41 (0.34–0.48)
**Equivalized incomes**					
**First quintile**				1 (reference)	1 (reference)
**Second quintile**				0.94 (0.83–1.06)	0.94 (0.83–1.06)
**Third quintile**				0.95 (0.82–1.09)	0.95 (0.82–1.09)
**Fourth quintile**				1.02 (0.89–1.17)	1.02 (0.89–1.17)
**Fifth quintile**				0.85 (0.74–0.98)	0.85 (0.74–0.98)
**Years of education**					
**≤ 6**				1 (reference)	1 (reference)
**7–9**				0.82 (0.70–0.96)	0.82 (0.70–0.96)
**10–12**				0.91 (0.79–1.04)	0.91 (0.79–1.04)
**≥ 13**				0.61 (0.52–0.71)	0.61 (0.53–0.71)
**Living with a partner**				0.91 (0.81–1.01)	0.91 (0.81–1.01)
**Districts level**					
**N. of primary clinics with good adequacy per 10,000**					1.10 (0.89–1.36)
**Random effects**					
**ICC**	0.020	0.021	0.020	0.020	0.041
**MOR**	1.28	1.29	1.28	1.28	1.43

All values were presented as “odds ratio (95% confidence interval)”.

[1]Walking for more than or equal to 30 min on at least 5 days per week.

ICC: intra-class coefficient correlation; MOR: median odds ratio.

Table S2. Multilevel multivariate logistic regression for obesity in diabetes patients

	Model 0	Model 1	Model 2	Model 3	Model 4
**Fixed effect**					
**Individuals level**					
**Age (10 years)**		0.79 (0.77–0.81)	0.77 (0.75–0.79)	0.73 (0.71–0.76)	0.74 (0.72–0.76)
**Women**		1.18 (1.11–1.24)	1.19 (1.09–1.29)	1.11 (1.01–1.21)	1.11 (1.02–1.21)
**Smoking history**					
**Never-smoker**			1 (reference)	1 (reference)	1 (reference)
**Ever-smoker**			1.16 (1.06–1.26)	1.16 (1.06–1.27)	1.16 (1.07–1.27)
**Current smoker**			0.76 (0.69–0.84)	0.75 (0.68–0.83)	0.75 (0.69–0.83)
**Drinking behavior**					
**Lifetime abstainers**			1 (reference)	1 (reference)	1 (reference)
**Ex-drinkers**			0.95 (0.87–1.04)	0.95 (0.88–1.04)	0.95 (0.87–1.04)
**Occasional drinkers**			0.96 (0.88–1.06)	0.98 (0.89–1.08)	0.97 (0.88–1.07)
**Current drinkers**			1.04 (0.95–1.13)	1.06 (0.97–1.15)	1.05 (0.96–1.14)
**Walking activity**[1]			0.84 (0.79–0.88)	0.84 (0.79–0.89)	0.84 (0.79–0.89)
**Poor self-rated health**			1.01 (0.98–1.04)	0.99 (0.96–1.03)	0.99 (0.96–1.03)
**Equivalized incomes**					
**First quintile**				1 (reference)	1 (reference)
**Second quintile**				1.00 (0.92–1.08)	1.00 (0.92–1.08)
**Third quintile**				0.96 (0.88–1.05)	0.96 (0.88–1.05)
**Fourth quintile**				1.08 (0.98–1.18)	1.08 (0.99–1.18)
**Fifth quintile**				0.96 (0.87–1.05)	0.96 (0.87–1.05)
**Years of education**					
**≤ 6**				1 (reference)	1 (reference)
**7–9**				0.79 (0.72–0.86)	0.79 (0.73–0.87)
**10–12**				0.71 (0.65–0.77)	0.71 (0.66–0.77)
**≥ 13**				0.75 (0.68–0.83)	0.76 (0.69–0.84)
**Living with a partner**				0.97 (0.91–1.04)	0.97 (0.91–1.04)
**Districts level**					
**N. of primary clinics with good adequacy per 10,000**					0.90 (0.78–1.04)
**Random effects**					
**ICC**	0.010	0.009	0.008	0.008	0.008
**MOR**	1.18	1.18	1.17	1.17	1.17

All values were presented as “odds ratio (95% confidence interval)”.

[1]Walking for more than or equal to 30 min on at least 5 days per week.

ICC: intra-class coefficient correlation; MOR: median odds ratio.

Table S3. Multilevel multivariate logistic regression for engaging in walking activity in diabetes patients

	Model 0	Model 1	Model 2	Model 3	Model 4
**Fixed effect**					
**Individuals level**					
**Age (10 years)**		0.99 (0.96–1.01)	0.98 (0.95–1.00)	0.98 (0.96–1.01)	0.98 (0.96–1.01)
**Women**		0.85 (0.80–0.90)	0.86 (0.79–0.94)	0.90 (0.82–0.98)	0.90 (0.82–0.98)
**Smoking history**					
**Never-smoker**			1 (reference)	1 (reference)	1 (reference)
**Ever-smoker**			1.04 (0.95–1.13)	1.05 (0.96–1.14)	1.05 (0.96–1.14)
**Current smoker**			0.82 (0.74–0.90)	0.84 (0.77–0.93)	0.84 (0.76–0.92)
**Drinking behavior**					
**Lifetime abstainers**			1 (reference)	1 (reference)	1 (reference)
**Ex-drinkers**			0.92 (0.84–1.00)	0.92 (0.84–1.00)	0.92 (0.84–1.01)
**Occasional drinkers**			1.04 (0.94–1.14)	1.03 (0.93–1.13)	1.04 (0.94–1.15)
**Current drinkers**			0.94 (0.86–1.03)	0.93 (0.85–1.02)	0.94 (0.86–1.03)
**Poor self-rated health**			0.74 (0.72–0.77)	0.75 (0.72–0.78)	0.75 (0.72–0.78)
**Body mass index (kg/m^2)**					
**< 18.5**			0.66 (0.53–0.82)	0.66 (0.54–0.82)	0.66 (0.53–0.81)
**18.5–22.9**			1 (reference)	1 (reference)	1 (reference)
**23.0–24.9**			0.87 (0.80–0.94)	0.87 (0.81–0.94)	0.87 (0.80–0.94)
**25.0–29.9**			0.80 (0.75–0.86)	0.80 (0.75–0.86)	0.80 (0.75–0.86)
**≥ 30.0**			0.63 (0.57–0.71)	0.64 (0.57–0.72)	0.64 (0.57–0.72)
**Equivalized incomes**					
**First quintile**				1 (reference)	1 (reference)
**Second quintile**				1.01 (0.94–1.10)	1.01 (0.93–1.10)
**Third quintile**				1.14 (1.04–1.25)	1.14 (1.04–1.24)
**Fourth quintile**				0.95 (0.87–1.04)	0.96 (0.87–1.05)
**Fifth quintile**				0.98 (0.89–1.08)	0.98 (0.89–1.08)
**Years of education**					
**≤ 6**				1 (reference)	1 (reference)
**7–9**				1.12 (1.03–1.23)	1.12 (1.02–1.22)
**10–12**				0.96 (0.88–1.04)	0.95 (0.88–1.04)
**≥ 13**				1.14 (1.03–1.26)	1.14 (1.03–1.26)
**Living with a partner**				1.07 (1.00–1.14)	1.07 (1.00–1.14)
**Districts level**					
**N. of primary clinics with good adequacy per 10,000**					1.39 (1.10–1.76)
**Random effects**					
**ICC**	0.038	0.038	0.038	0.038	< 0.001
**MOR**	1.41	1.41	1.41	1.41	1.00

All values were presented as “odds ratio (95% confidence interval)”.

[1]Walking for more than or equal to 30 min on at least 5 days per week.

ICC: intra-class coefficient correlation; MOR: median odds ratio.

Table S4. Multilevel multivariate logistic regression for fundus examination annually in diabetes patients

	Model 0	Model 1	Model 2	Model 3	Model 4
**Fixed effect**					
**Individuals level**					
**Age (10 years)**		1.03 (1.01–1.05)	0.99 (0.96–1.01)	1.09 (1.06–1.12)	1.09 (1.06–1.12)
**Women**		1.16 (1.09–1.22)	1.00 (0.92–1.09)	1.17 (1.07–1.28)	1.17 (1.07–1.28)
**Smoking history**					
**Never-smoker**			1 (reference)	1 (reference)	1 (reference)
**Ever-smoker**			0.99 (0.90–1.08)	0.99 (0.91–1.08)	1.00 (0.91–1.09)
**Current smoker**			0.72 (0.65–0.79)	0.76 (0.69–0.83)	0.76 (0.69–0.83)
**Drinking behavior**					
**Lifetime abstainers**			1 (reference)	1 (reference)	1 (reference)
**Ex-drinkers**			1.00 (0.91–1.09)	1.01 (0.92–1.10)	1.01 (0.92–1.10)
**Occasional drinkers**			1.08 (0.98–1.19)	1.07 (0.97–1.17)	1.07 (0.97–1.18)
**Current drinkers**			0.93 (0.85–1.02)	0.91 (0.83–0.99)	0.91 (0.84–1.00)
**Physical activity[1]**			1.20 (1.13–1.27)	1.20 (1.13–1.27)	1.20 (1.13–1.27)
**Self-rated health**			1.16 (1.12–1.20)	1.21 (1.17–1.25)	1.21 (1.17–1.25)
**Body mass index (kg/m^2)**					
**< 18.5**			1 (reference)	1 (reference)	1 (reference)
**18.5–22.9**			0.86 (0.70–1.06)	0.90 (0.73–1.11)	0.91 (0.74–1.12)
**23.0–24.9**			1.07 (0.99–1.15)	1.08 (1.00–1.17)	1.09 (1.00–1.17)
**25.0–29.9**			0.93 (0.87–1.00)	0.95 (0.89–1.02)	0.95 (0.89–1.02)
**≥ 30.0**			0.88 (0.79–0.98)	0.92 (0.82–1.03)	0.93 (0.83–1.03)
**Equivalized incomes**					
**First quintile**				1 (reference)	1 (reference)
**Second quintile**				1.08 (0.99–1.17)	1.08 (0.99–1.17)
**Third quintile**				1.00 (0.91–1.10)	1.00 (0.91–1.10)
**Fourth quintile**				1.11 (1.01–1.22)	1.11 (1.01–1.22)
**Fifth quintile**				1.34 (1.21–1.47)	1.34 (1.21–1.48)
**Years of education**					
**≤ 6**				1 (reference)	1 (reference)
**7–9**				1.24 (1.13–1.36)	1.24 (1.13–1.36)
**10–12**				1.50 (1.38–1.63)	1.49 (1.37–1.63)
**≥ 13**				1.61 (1.45–1.78)	1.61 (1.45–1.78)
**Living with a partner**				1.14 (1.06–1.21)	1.14 (1.07–1.21)
**Districts level**					
**N. of primary clinics with good adequacy per 10,000**					1.33 (1.05–1.68)
**Random effects**					
**ICC**	0.041	0.041	0.041	0.037	0.008
**MOR**	1.43	1.43	1.43	1.40	1.16

All values were presented as “odds ratio (95% confidence interval)”.

[1]Walking for more than or equal to 30 min on at least 5 days per week.

ICC: intra-class coefficient correlation; MOR: median odds ratio.

Table S5. Multilevel multivariate logistic regression for microalbuminuria test annually in diabetes patients

	Model 0	Model 1	Model 2	Model 3	Model 4
**Fixed effect**					
**Individuals level**					
**Age (10 years)**		0.97 (0.94–0.99)	0.93 (0.91–0.95)	1.00 (0.97–1.03)	1.00 (0.97–1.03)
**Women**		1.04 (0.98–1.10)	0.98 (0.90–1.07)	1.11 (1.02–1.22)	1.12 (1.02–1.22)
**Smoking history**					
**Never-smoker**			1 (reference)	1 (reference)	1 (reference)
**Ever-smoker**			1.08 (0.99–1.19)	1.08 (0.99–1.19)	1.09 (0.99–1.19)
**Current smoker**			0.87 (0.79–0.96)	0.91 (0.82–1.00)	0.91 (0.83–1.00)
**Drinking behavior**					
**Lifetime abstainers**			1 (reference)	1 (reference)	1 (reference)
**Ex-drinkers**			1.01 (0.92–1.10)	1.01 (0.92–1.10)	1.01 (0.92–1.11)
**Occasional drinkers**			0.97 (0.88–1.07)	0.96 (0.87–1.06)	0.96 (0.87–1.06)
**Current drinkers**			0.88 (0.80–0.96)	0.85 (0.78–0.93)	0.86 (0.78–0.94)
**Physical activity[1]**			1.10 (1.04–1.16)	1.10 (1.04–1.16)	1.10 (1.04–1.16)
**Self-rated health**			1.12 (1.08–1.16)	1.15 (1.11–1.19)	1.15 (1.11–1.19)
**Body mass index (kg/m^2)**					
**< 18.5**			1 (reference)	1 (reference)	1 (reference)
**18.5–22.9**			0.74 (0.60–0.92)	0.78 (0.63–0.96)	0.78 (0.63–0.96)
**23.0–24.9**			1.08 (1.00–1.17)	1.09 (1.01–1.18)	1.09 (1.01–1.18)
**25.0–29.9**			0.96 (0.89–1.03)	0.98 (0.91–1.05)	0.98 (0.91–1.05)
**≥ 30.0**			0.94 (0.84–1.05)	0.98 (0.88–1.10)	0.98 (0.88–1.10)
**Equivalized incomes**					
**First quintile**				1 (reference)	1 (reference)
**Second quintile**				1.06 (0.98–1.15)	1.06 (0.97–1.14)
**Third quintile**				0.96 (0.88–1.05)	0.96 (0.88–1.05)
**Fourth quintile**				1.04 (0.94–1.14)	1.04 (0.94–1.14)
**Fifth quintile**				1.31 (1.19–1.45)	1.32 (1.19–1.45)
**Years of education**					
**≤ 6**				1 (reference)	1 (reference)
**7–9**				1.14 (1.04–1.24)	1.13 (1.04–1.24)
**10–12**				1.35 (1.24–1.47)	1.35 (1.24–1.47)
**≥ 13**				1.38 (1.25–1.53)	1.38 (1.25–1.53)
**Living with a partner**				1.19 (1.12–1.27)	1.20 (1.12–1.28)
**Districts level**					
**N. of primary clinics with good adequacy per 10,000**					1.46 (1.13–1.87)
**Random effects**					
**ICC**	0.065	0.064	0.065	0.062	0.031
**MOR**	1.57	1.57	1.57	1.55	1.36

All values were presented as “odds ratio (95% confidence interval)”.

[1]Walking for more than or equal to 30 min on at least 5 days per week.

ICC: intra-class coefficient correlation; MOR: median odds ratio.

Table S6. Multilevel multivariate logistic regression for HbA1c test twice a year in diabetes patients

	Model 0	Model 1	Model 2	Model 3	Model 4
**Fixed effect**					
**Individuals level**					
**Age (10 years)**		0.75 (0.74–0.77)	0.73 (0.71–0.75)	0.86 (0.83–0.88)	0.86 (0.83–0.89)
**Women**		0.96 (0.90–1.02)	0.98 (0.90– 1.07)	1.27 (1.16–1.39)	1.27 (1.16–1.40)
**Smoking history**					
**Never-smoker**			1 (reference)	1 (reference)	1 (reference)
**Ever-smoker**			1.22 (1.11– 1.34)	1.24 (1.13–1.37)	1.24 (1.13–1.37)
**Current smoker**			0.81 (0.74– 0.90)	0.88 (0.80–0.98)	0.89 (0.80–0.98)
**Drinking behavior**					
**Lifetime abstainers**			1 (reference)	1 (reference)	1 (reference)
**Ex-drinkers**			1.22 (1.11– 1.34)	1.24 (1.13–1.37)	1.24 (1.13–1.37)
**Occasional drinkers**			0.81 (0.74– 0.90)	0.88 (0.80–0.98)	0.89 (0.80–0.98)
**Current drinkers**			0.97 (0.88– 1.06)	0.97 (0.89–1.07)	0.98 (0.89–1.07)
**Physical activity[1]**			1.10 (1.00– 1.22)	1.08 (0.98–1.20)	1.09 (0.98–1.21)
**Self-rated health**			0.97 (0.88– 1.06)	0.93 (0.84–1.01)	0.93 (0.85–1.02)
**Body mass index (kg/m^2)**					
**< 18.5**			1 (reference)	1 (reference)	1 (reference)
**18.5–22.9**			0.97 (0.79– 1.21)	1.05 (0.85–1.31)	1.06 (0.85–1.31)
**23.0–24.9**			1.05 (0.97– 1.14)	1.08 (1.00–1.17)	1.08 (1.00–1.17)
**25.0–29.9**			0.97 (0.90– 1.04)	1.00 (0.93–1.08)	1.01 (0.93–1.08)
**≥ 30.0**			0.91 (0.81– 1.02)	0.98 (0.87–1.10)	0.99 (0.88–1.11)
**Equivalized incomes**					
**First quintile**				1 (reference)	1 (reference)
**Second quintile**				1.14 (1.05–1.24)	1.14 (1.05–1.24)
**Third quintile**				1.44 (1.31–1.58)	1.44 (1.31–1.58)
**Fourth quintile**				1.21 (1.09–1.33)	1.21 (1.10–1.33)
**Fifth quintile**				1.43 (1.29–1.59)	1.43 (1.29–1.59)
**Years of education**					
**≤ 6**				1 (reference)	1 (reference)
**7–9**				1.40 (1.28–1.53)	1.40 (1.28–1.53)
**10–12**				1.90 (1.74–2.07)	1.90 (1.75–2.07)
**≥ 13**				2.27 (2.04–2.52)	2.28 (2.05–2.53)
**Living with a partner**				1.27 (1.19–1.36)	1.27 (1.19–1.36)
**Districts level**					
**N. of primary clinics with good adequacy per 10,000**					1.38 (1.08–1.76)
**Random effects**					
**ICC**	0.062	0.067	0.066	0.057	0.044
**MOR**	1.56	1.58	1.58	1.53	1.45

All values were presented as “odds ratio (95% confidence interval)”.

[1]Walking for more than or equal to 30 min on at least 5 days per week.

HbA1c: glycated hemoglobin; ICC: intra-class coefficient correlation; MOR: median odds ratio.
